# In Acute IgA Nephropathy, Proteinuria and Creatinine Are in the Spot, but Podocyturia Operates in Silence: Any Place for Amiloride?

**DOI:** 10.1155/2017/1292531

**Published:** 2017-04-03

**Authors:** H. Trimarchi, M. Paulero, R. Canzonieri, A. Schiel, A. Iotti, C. Costales-Collaguazo, A. Stern, M. Forrester, F. Lombi, V. Pomeranz, R. Iriarte, T. Rengel, I. Gonzalez-Hoyos, A. Muryan, E. Zotta

**Affiliations:** ^1^Nephrology, Hospital Británico de Buenos Aires, Buenos Aires, Argentina; ^2^Biochemistry, Hospital Británico de Buenos Aires, Buenos Aires, Argentina; ^3^Pathology Services, Hospital Británico de Buenos Aires, Buenos Aires, Argentina; ^4^IFIBIO Houssay-UBA CONICET, Facultad de Medicina, Universidad de Buenos Aires, Argentina

## Abstract

IgA nephropathy is the most frequent cause of primary glomerulonephritis, portends erratic patterns of clinical presentation, and lacks specific treatment. In general, it slowly progresses to end-stage renal disease. The clinical course and the response to therapy are usually assessed with proteinuria and serum creatinine. Validated biomarkers have not been identified yet. In this report, we present a case of acute renal injury with proteinuria and microscopic hematuria in a young male. A kidney biopsy disclosed IgA nephropathy. Podocyturia was significantly elevated compared to normal subjects. Proteinuria, renal function, and podocyturia improved promptly after steroids and these variables remained normal after one year of follow-up, when steroids had already been discontinued and patient continued on valsartan and amiloride. Our report demonstrates that podocyturia is critically elevated during an acute episode of IgA nephropathy, and its occurrence may explain the grim long-term prognosis of this entity. Whether podocyturia could be employed in IgA nephropathy as a trustable biomarker for treatment assessment or even for early diagnosis of IgA nephropathy relapses should be further investigated.

## 1. Introduction

IgA nephropathy is the most frequent cause of primary glomerulopathies worldwide. It can clinically emerge with a wide variety of presentations, ranging from asymptomatic microhematuria to nephrotic syndrome or rapidly progressive glomerulonephritis. It is an autoimmune disease diagnosed by immunofluorescent positive IgA mesangial deposits in the kidney biopsy [1]. Hypogalactosylated IgA molecules evoke the synthesis of IgG autoantibodies that form circulating immune complexes that end up entrapped in the mesangial area due to interactions between CD89 and mesangial cell receptors like CD71. Finally, this abnormal deposition of immune complexes in the glomerulus causes local inflammation, mesangial proliferation, matrix expansion, and eventually fibrosis, while endocapillary proliferation and extracapillary proliferation are more rare findings [2]. All these histologic alterations correlate with hematuria and proteinuria, the latter being the main culprit of chronic kidney disease progression. Finally, protein trafficking in the kidney interstitium results in interstitial fibrosis and tubular atrophy. Renal function decline is mainly due to glomerular obliteration and interstitial changes, which generally occur in a chronic progressive course.

The only usual markers that physicians count on to assess the above morphologic derangements in routine clinical practice are proteinuria, dysmorphic hematuria, and creatinine creeping. Like in most glomerulopathies, in IgA nephropathy, there are no specific validated biomarkers. When matrix expansion is observed in a glomerulus in the kidney biopsy, it is estimated that approximately 20% of the podocyte population of that glomerulus has already been detached and forever lost in the urine, as podocytes are not capable of undergoing mitosis [3, 4]. This phenomenon is named podocyturia [4]. When the glomerulus loses between 20 and 40% of the podocyte population, usually associated with morphologic changes as focal adhesions between the glomerular tuft and Bowman capsule and/or focal and segmental glomerulosclerosis, it is rendered to be obliterated [3]. We present a case of acute renal failure with proteinuria and microhematuria in a young male whose biopsy disclosed IgA nephropathy. Concurrent significant podocyturia was observed. After immunosuppressive therapy, proteinuria became negative, renal function was recovered, and podocyturia remained similar to controls. When renal function improved, valsartan and amiloride were prescribed and steroids discontinued. We believe that the loss of podocytes in IgA nephropathy could be the reason why this entity shows a relentless progressive course in most of these patients. Podocyturia could be employed to guide the type of therapy to be employed and also as a follow-up tool for nephrologists.

## 2. Case Presentation

**Figure 1:**
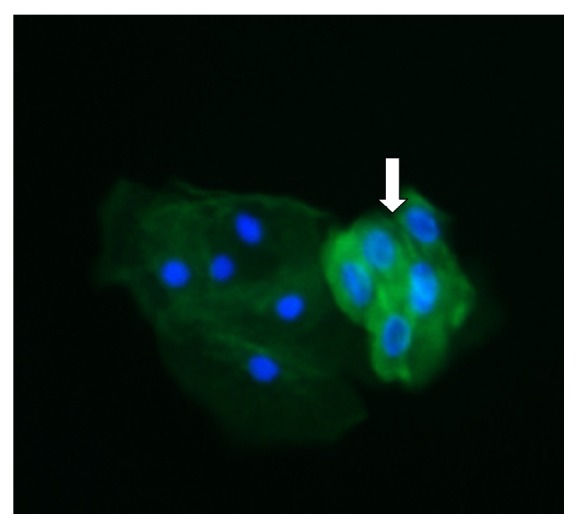
The white arrow indicates the presence of clustered podocytes, as bright green fluorescent cells. A tubular cell is observed to the left. Fluorescent microscopy, ×200.

A healthy 18-year-old male was admitted due to kidney failure (serum creatinine: 3 mg/dL), proteinuria (1.89 g/day), and recent past episodes of intermittent macrohematuria. Urine smear disclosed 30–50 red blood cells per high power field (Table 1). Four months before admission, a routine blood exam had been within normal limits, except for a urinalysis that had shown 10 red blood cells per high power field. Past medical history was negative for prior upper respiratory tract infections or any infections of clinical relevance. At the time of admission, physical examination was normal; blood pressure was 110/70 mmHg. A renal ultrasound showed kidneys with normal shape and measurements with echogenic alterations, while all complementary serologic studies were noncontributory: blood, urine, and throat cultures, HIV, hepatitis B and C serologies, complement levels, p- and c-ANCA antibodies, antiglomerular basement membrane antibodies, and electrophoretic proteinogram. Podocyturia was performed as described previously [5]. Briefly, the podocyte count was assessed by counting in urinary smears the number of cells in 10 microscopy fields of ×20. The podocyte count was 2.1 cells per ×20 field and the number of podocytes per gram of urinary creatinine was 166 (Figure 1). Podocytes were identified by tagging synaptopodin (ab109560 Alexa Fluor®, Abcam, Cambridge, UK), an specific marker of podocytes, to establish their identity by immunofluorescence techniques using a secondary antibody (Alexa Fluor 488, Abcam, Cambridge, Uk). The smears were analyzed employing an epifluorescence microscopy, Nikon Eclipse E200. This result was compared with 10 controls (6 males and 4 females; mean age: 20 ± 2.1 years, with no past history of morbidities: serum creatinine, 0.7 ± 0.1 mg/dL; mean 24-hour urinary albumin excretion, 66 ± 12 mg/day. The mean podocyte count was 0.11 ± 0.3 cells per ×20 field, while the mean number of podocytes per gram of urinary creatinine was 8.3. One day after admission, a kidney biopsy was performed and was consistent with IgA nephropathy, with the following Oxford score over 14 glomeruli: M1E0S0T0. The immunofluorescence was positive for IgA and mesangial C3 deposits +++/4 y C3. Due to the rapidly progressive course of the altered kidney function, oral methyl prednisone 1 mg/kg/day was started. Two weeks later when creatinine was 1.2 mg/dL, amiloride was started at 5 mg/day. Four weeks after initiation of therapy, proteinuria and hematuria were negative and serum creatinine dropped to 0.7 mg/dL (Table 1). Two consecutive podocyturia tests were similar to controls after one year of follow-up. Current therapy consists of amiloride 5 mg/day and valsartan 80 mg/day.

## 3. Discussion

In our opinion, this case report illustrates several aspects of IgA nephropathy which deserve special consideration. From the clinical point of view, an acute episode of renal injury due to IgA nephropathy may not necessarily be associated with endocapillary proliferation or extracapillary proliferation. However, it must be borne in mind that, due to limitations inherent to a biopsy sample size factor, areas of endocapillary proliferation or extracapillary proliferation may have well been missed. In addition, in IgA nephropathy, endocapillary proliferation is not easily identified, even by trained pathologists [6]. Although the previous episodes of macrohematuria might have well contributed to transient creeping of serum creatinine, as described previously [7], creatinine continued to rise long after macrohematuria had disappeared.

Certainly, a prompt therapeutic intervention may have stopped the potential development of a more proliferative histologic pattern. Macroscopic hematuria was the one sign that alerted the patient for a quick medical consult. In this regard, it is interesting to contrast our findings with the ones published by Asao et al. who reported the relationships between podocyturia marked with podocalyxin, the level of urinary podocalyxin, and the histologic findings in 51 patients with IgA nephropathy [8]. Our urinary podocyte number adjusted to grams of urinary creatinine was lower when compared to their results, and a control cohort of 10 patients was employed. This observation could have many explanations: our Oxford score was only positive for M1, while their patients included many different Oxford scores. In addition, their findings underscored the noteworthy observation that higher levels of podocyturia correlated with glomerulosclerosis and extracapillary proliferation, histologic findings that our patient did not present. In addition, our case represents an early clinical event, while their work included 51 patients, many with advanced lesions, and no control group was included. Finally, different markers were employed, which assess different podocyte compartments. Asao et al. have clearly stated that podocalyxin, a sialomucin that is located in the glycocalyx, is not specific for podocytes [8]. Synaptopodin is a postmitotic cytoplasmic podocyte protein that has been proven by many authors to be a reliable and specific marker of podocytes [5, 9, 10]. As Maestroni et al. have shown and as we have addressed in our previous publications, there are different podocyte subpopulations that may be missed if only one biomarker is employed, as is the case in our case report and Asato's elegant work [4, 5, 9].

Advantages and disadvantages of measuring podocyturia emerge in clinical practice. As we have previously reported, the methods employed to assess podocyturia are time-consuming, are laborious, and must be performed by skilled professionals [5]. However, it offers many advantages as a biomarker for early stages of glomerular injury and can be employed to unravel the pathophysiologic pathways of podocyte detachment [submitted work]. We believe that as detached podocytes may be released intermittently, serial podocyturia employing several markers would be a more reliable way to assess podocyte loss (group ongoing study).

Another controversial aspect of the present case is the therapy employed. As the patient was with renal failure and serum creatinine worsened after admission until the biopsy was performed, renin-angiotensin blockade was not initiated. As proteinuria was >1 g/day, the patient was started on steroids [11, 12]. However, this prescription may be questioned by the fact that the Oxford score, based on a validated histologic classification with proposed standardization of diagnosis and also with impact in clinical outcomes, was low in aggressiveness [13]. Moreover, in the recently published STOP-IGAN trial, the addition of immunosuppressants to renin-angiotensin blockade and supportive care would not provide substantial kidney-related benefits in patients with high-risk IgA nephropathy, due to the fact that there are no differences in the rate of decrease in renal function, although corticosteroid/immunosuppressive therapy induced complete remission of proteinuria more frequently than supportive care alone. Side effects were more common in those who had received immunosuppression. Finally, immunosuppressed patients had a worse outcome than those treated with renin-angiotensin inhibition. Noteworthy, in this important prospective study, kidney biopsies were not included [14]. Recent data support that patients with preserved renal function and proteinuria > 1 g/day or subjects with E1 lesions or with apparent isolated nonaggressive M1 lesion in the Oxford score plus proteinuria < 0.75 g/day may benefit from steroid therapy [11]. Steroids were indicated due to acute renal injury in a young adult with >1 gram of proteinuria. In our opinion, as has been shown by numerous studies, we believe that there is no question that angiotensin converting enzyme inhibitors and/or angiotensin receptor blockers are the first-line drugs to be employed in IgA nephropathy patients. However, from the physiopathological point of view, we treated the patient with amiloride, as podocyturia was elevated, due to the fact that we and others have demonstrated that, in the podocyte detachment that occurs in IgA nephropathy, podocyte urokinase-type plasminogen activator receptor (uPAR) may be involved, with potential coupling of basal membrane integrins, such as *α*V*β*3 or *α*3*β*1 [15]. We have also assessed the use of amiloride without steroids in patients with other glomerulopathies, and amiloride was successful to decrease podocyturia in the long term as maintenance therapy [16, 17]. Amiloride blocks the synthesis of uPAR and the consequent coupling to *β* subunits of integrins [18]. This would result in a decreased interaction of uPAR with podocyte actin and with integrins, diminishing podocyte contraction and motility and the risk of detachment and proteinuria [15–18]. In summary, the rationale of our therapeutic approach was to start the patient on amiloride first so as to decrease the podocyte loss, and after a more clinical steady state, valsartan, a proven drug that improves IgA nephropathy prognosis, was added [12, 16, 17]. It could be argued that amiloride ought not to have been prescribed. However, as the patient was hemodynamically stable, no hyperkalemia was present, and serum creatinine had decreased from 3 mg/dL to 1.2 mg/dl after two weeks, we assumed the initial creatinine creep to be due to IgA nephropathy activity, based on the urinary findings, clinical picture, and presumably also the steroid response. In this setting, amiloride could be employed at the acute phase of the disease to potentially intervene on podocyte loss, as addressed above.

We believe that if podocyturia is identified, targeted, and decreased, particularly at early stages of a glomerulopathy as IgA nephropathy, the consequent progression of these entities to chronic kidney disease may be at least delayed. In this respect, IgA nephropathy lacks any specific treatment [19]. Podocyturia has been previously addressed in IgA nephropathy [15, 19, 20]. Hara et al. have demonstrated that podocyte loss reflected disease progression [20]. Renin-angiotensin blockade plays a critical role in nephroprotection in IgA nephropathy [11, 12, 14]. This benefit may be due to the effects angiotensin converting enzyme inhibitors and angiotensin receptor blockers exert not only on the interference of angiotensin actions but also on the stabilization they play on the podocyte [21]. The identification of validated biomarkers for the early identification of this disease or for follow-up purposes is mandatory in IgA nephropathy. Podocyturia could be an available tool to count on in patients with this progressive disease. Amiloride is a drug that could be assessed in clinical trials in IgA nephropathy based on the role podocyturia plays in IgA nephropathy and the potential inhibition of podocyte detachment on this entity.

## Figures and Tables

**Table 1:** Clinical, biochemical, and interventional data.

Variables	Days							
	0	1	2	3	4	15	30	365
	Interventions							
	Admission	Biopsy steroids		Biopsy result	Hospital discharge	Amiloride	Valsartan	
Blood pressure (mmHg)	110/70	124/76	120/70	120/72	110/76	126/72	116/78	110/66
Serum creatinine	3	3.1	2.6	2.2	1.6	1.2	0.7	0.9
CKD-EPI (mL/min)	29	28	34	42	62	88	138	124
Podocytes/gram urinary creatinine (cells/g)	166	NP	NP	NP	NP	110	0.8	0.7
Proteinuria (g/day)	1.89	NP	NP	NP	1.1	0.5	0.07	0.09
Dysmorphic red blood (%)	95	NP	NP	NP	89	40	15	20
Serum potassium (mEq/L)	4.9	4.8	4.3	4.1	3.8	3.7	4.1	4.3

NP, not performed.
